# Establishment of a model for equine small intestinal disease: effects of extracorporeal blood perfusion of equine ileum on metabolic variables and histological morphology – an experimental ex vivo study

**DOI:** 10.1186/s12917-019-2145-9

**Published:** 2019-11-08

**Authors:** Maria S. Unterköfler, Bruce C. McGorum, Elspeth M. Milne, Theresia F. Licka

**Affiliations:** 1 Department for Companion Animals and Horses, University Equine Hospital, University of Veterinary Medicine Vienna, Veterinärplatz 1, 1210 Vienna, Austria; 2 Department of Veterinary Clinical Sciences, Royal (Dick) School of Veterinary Studies and The Roslin Institute, The University of Edinburgh, Easter Bush Campus, Midlothian, EH25 9RG UK

**Keywords:** Dysautonomia, Extracorporeal perfusion, Horse, Ileum, Grass sickness, Ileus, Reperfusion injury

## Abstract

**Background:**

In horses a number of small intestinal diseases is potentially life threatening. Among them are Equine Grass Sickness (EGS), which is characterised by enteric neurodegeneration of unknown aetiology, as well as reperfusion injury of ischaemic intestine (I/R), and post-operative ileus (POI), common after colic surgery. The perfusion of isolated organs is successfully used to minimize animal testing for the study of pathophysiology in other scenarios. However, extracorporeal perfusion of equine ileum sourced from horses slaughtered for meat production has not yet been described. Therefore the present study evaluated the potential of such a model for the investigation of small intestinal diseases in an ex vivo and cost-efficient system avoiding experiments in live animals.

**Result:**

Nine ileum specimens were sourced from horses aged 1–10 years after routine slaughter at a commercial abattoir. Ileum perfusion with oxygenated autologous blood and plasma was successfully performed for 4 h in a warm isotonic bath (37.0–37.5 °C). Ileum specimens had good motility and overall pink to red mucosa throughout the experiment; blood parameters indicated good tissue vitality: 82 ± 34 mmHg mean arterial partial pressure of oxygen (pO_2_) compared to 50 ± 17 mmHg mean venous pO_2,_ 48 ± 10 mmHg mean arterial partial pressure of carbon dioxide (pCO_2_) compared to 66 ± 7 mmHg venous pCO_2_ and 9.8 ± 2.8 mmol/L mean arterial lactate compared to 11.6 ± 2.7 mmol/L venous lactate. There was a mild increase in ileum mass reaching 105 ± 7.5% of the pre-perfusion mass after 4 hours. Histology of haematoxylin and eosin stained biopsy samples taken at the end of perfusion showed on average 99% (±1%) histologically normal neurons in the submucosal plexus and 76.1% (±23.9%) histologically normal neurons in the myenteric plexus and were not significantly different to control biopsies.

**Conclusion:**

Extracorporeal, normothermic perfusion of equine ileum over 4 h using autologous oxygenated blood/plasma perfusate showed potential as experimental model to test whether haematogenous or intestinal exposure to neurotoxins suspected in the pathogenesis of EGS can induce neuronal damage typical for EGS. Also, this model may allow investigations into the effect of pharmaceuticals on I/R injury, as well as into the pathogenesis of equine POI.

## Background

Gastrointestinal diseases are the most common emergency in equine hospitals, with acute colic being the most frequent medical problem requiring surgery in up to 17.5% of cases [1]. Life threatening conditions such as Equine Grass Sickness (EGS), ischemia/reperfusion injury (I/R) or post-operative ileus (POI) require further investigation in aetiology, treatment and pathogenesis [1–4]. EGS, also known as Equine Dysautonomia, is a degenerative disease of the neuronal system in equids, of unknown aetiology, which was first described in 1907. There are reported cases all over the world, though the disease primarily occurs in the United Kingdom. Clinical signs and severity of the disease are largely defined by degeneration of neurons in the enteric nervous system. The disease almost exclusively appears in grazing horses and, although many agents are being discussed, the causative factor remains to be found [2]. Furthermore, intestinal volvulus and strangulation create ischemia of affected intestine and once necessary perfusion is surgically re-established, intestinal damage is often exacerbated. This problem, known as I/R injury, is characterized by generation of reactive oxygen metabolites, activation of immune cells, and epithelial cell degeneration and death. Efforts in pharmaceutical intervention have been made with ambiguous results [3]. After colic surgery, horses are also at risk of developing small intestinal POI, a serious condition of reduced or absent intestinal peristalsis without mechanical obstruction. Mechanisms of this disease, such as intestinal manipulation, disruption of neuronal or hormonal continuity, or effects of pharmaceutical interventions, are mainly proposed based on research in rodents and on its parallels to human ileus; however these results may not be directly related to the horse, as human ileus involves both small and large intestine [4].

Due to the severity of the described diseases it is be ethically preferable to study these conditions without using live horses in the first instance. The extracorporeal perfusion of organs from horses slaughtered for meat production offers an ethical method for studying a system similar to that of a living animal [5]. Reported models for extracorporeal perfusion of intestine in large animals are mainly based on pig small intestines [6–9], which were perfused for up to 160 min [9]. In horses, perfusion of the jejunum under general anaesthesia in live horses [10] and of the large colon from euthanized horses [11] has been performed. In these studies, live animals were used solely for the purpose of the study and, to the authors’ knowledge, no attempt has been made to use the intestines of animals slaughtered for meat production for extracorporeal perfusion. A successful model was developed using isolated equine limbs from slaughtered horses to investigate equine laminar tissue [12]. This model has been widely used for various purposes and delivered insight into the pathogenesis of laminitis on a very cost-efficient and ethically sound basis [13–16]. The present study is the first step toward establishing an extracorporeal ileum perfusion model to investigate EGS [17]; furthermore this model may later be developed to be used in, amongst others, intestinal hormonal studies, investigation of inflammatory bowel disease or as an animal model for organ transplantation in human medicine.

## Results

### Animals

Nine horses were selected as ileum donors. The group of horses consisted of 4 mares, 3 stallions and 2 geldings of various breeds, ranging from 1 year to 10 years of age (mean 5.67 ± 3.74 years), with a body mass from 400 kg to 700 kg (mean 511.11 ± 92.8 kg) and a body condition score from 4/9 to 5/9 (mean 4.67/9 ± 0.5).

### Perfusion

**Fig. 1:**
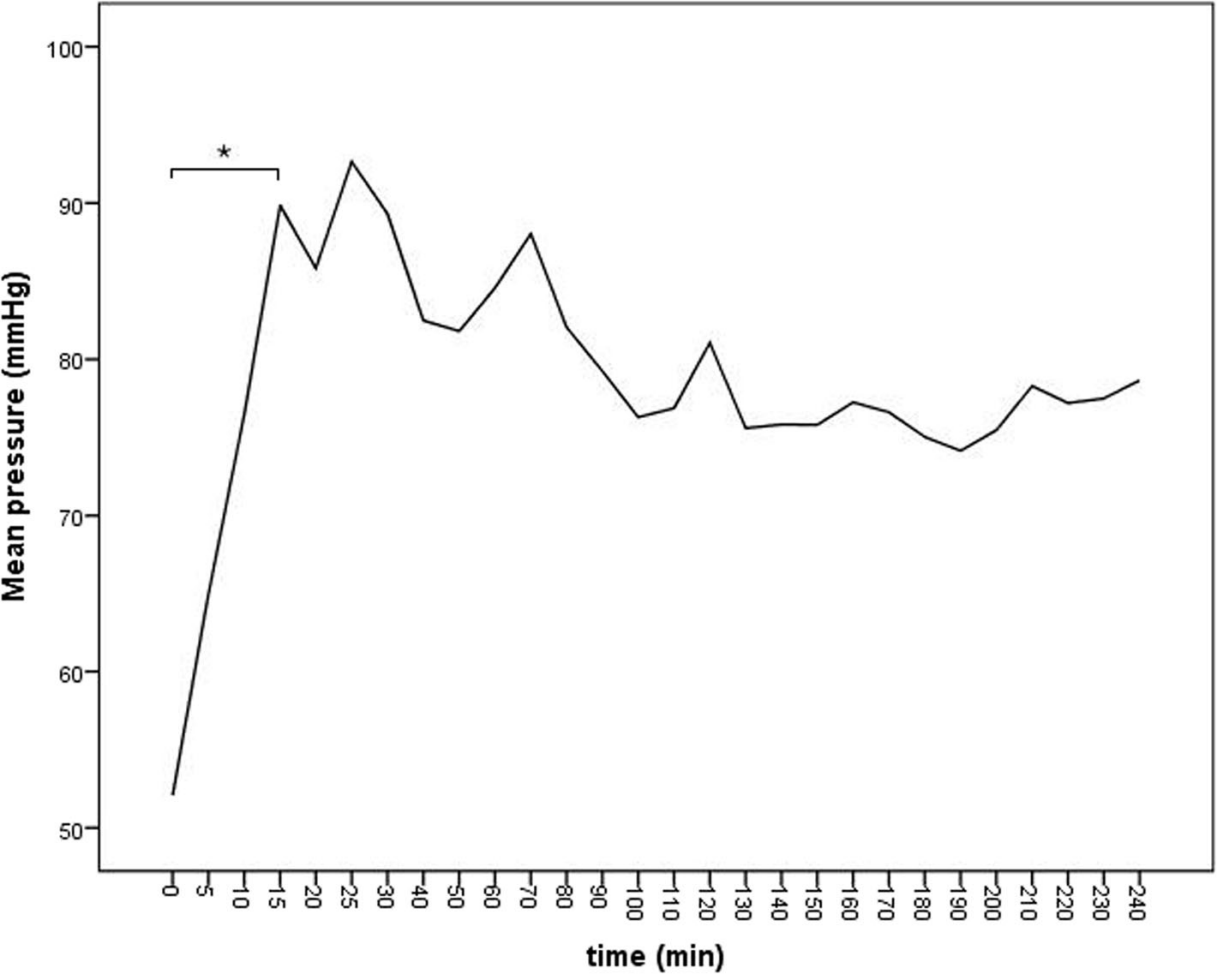
Blood pressure rose significantly to physiological values within the equilibration period (*P* < 0.001, bar with asterisk); from then until the end of equilibration period it remained stable

The time between slaughter and ileum isolation and cannulation, i.e. warm ischemia time was between 29 min and 51 min (mean 43.89 ± 6.19 min) and cold ischemia time (mainly transport) was between 186 min and 330 min (mean 219 ± 46.98 min). Blood flow and blood pressure after the equilibration period was between 7 mL/min and 40 mL/min (mean 21.33 ± 9.38 mL/min) and 29.4 mmHg and 137.2 mmHg (mean 78.56 ± 22.07 mmHg). Mean blood pressure over time is shown in Fig. 1. All specimens started distinct peristaltic motility after 5 min to 60 min of perfusion (mean 25 ± 16.6 min); this continued throughout the experiment. The colour of the serosa was pink at the start of the perfusion and changed from pink to reddish after 20 min to 90 min (mean 58.9 ± 26.2 min), while 4 specimens developed cyanotic discolouration after 200 min to 220 min (mean 207.5 ± 9.6 min). The perfusate loss of the specimen required the reservoir to be replenished with 0.5 L to 2.9 L (mean 1.3 ± 0.9 L) during the 4 h. Mass of the ilei after perfusion was between 94.4 and 116.3% (mean 105 ± 7.5%) of pre-perfusion mass.

### Perfusate samples

A descriptive summary of the analysed blood parameters is shown in Table 1, and significant differences from published reference intervals [18] are indicated.

**Table 1:** Summary of analysed blood parameters compared to reference intervals

	N	mean (SD)	median (range)		reference intervals by Corley, 2008
Tp v (g/L)	45	71 (7)	70 (61–85)		58–77
pH a	44	7.3 (0.09)	7.28 (7.1–7.43)	↓	7.36–7.44
pH v	43	7.18 (0.07)	7.18 (7.04–7.48)	↓	7.33–7.41
pCO_2_ a (mmHg)	43	48 (10)	44 (34–67)		37–49
pCO_2_ v (mmHg)	41	66 (7)	67 (50–79)	↑	46–64
pO_2_ a (mmHg)	40	82 (34)	70 (45–172)		89–115
pO_2_ v (mmHg)	39	49.5 (17)	46.2 (36.1–138.8)		28.7–48.6
HCO_3_^−^ a (mmol/L)	43	23 (2)	23 (19–27)		23–30
HCO_3_^−^ v (mmol/L)	41	23.1 (4.9)	23.6 (−5.5–29.9)	↓	31.6–37.7
BE a (mmol/L)	43	-4 (2)	−3 (−9–0)	↓	−2-2
BE v (mmol/L)	40	−4.8 (2)	−4.8 (− 9--1.3)	↓	6.4–12.3
PCV a (%)	42	27 (6)	28 (13–41)		24–53
PCV v (%)	41	25 (8)	24 (9–39)		24–53
Na^+^ a (mmol/L)	44	138.6 (3.8)	138.3 (131.6–152)		137.7–142
Na^+^ v (mmol/L)	43	143.6 (3.8)	143.5 (134.8–154)	↑	134.7–142
K^+^ a (mmol/L)	44	5.6 (0.65)	5.72 (4.46–6.95)	↑	3.53–4.64
K^+^ v (mmol/L)	43	5.99 (1.15)	5.6 (4.46–9.6)	↑	3.53–4.64
Ca^2+^ a (mmol/L)	42	1.51 (0.07)	1.51 (1.4–1.68)		1.45–1.73
Ca^2+^ v (mmol/L)	41	1.54 (0.07)	1.53 (1.42–1.71)		1.45–1.73
Cl^−^ a (mmol/L)	44	107.5 (3.1)	107.5 (101.3–114)	↑	97.3–103.6
Cl^−^ v (mmol/L)	43	109.4 (3.2)	110.4 (101.5–114.2)	↑	97.3–103.6
glucose a (mmol/L)	41	6.3 (1.6)	5.9 (4.2–10.6)		4.9–6.2
glucose v (mmol/L)	43	8.4 (2.4)	8.1 (5.6–18.9)	↑	4.9–6.2
lactate a (mmol/L)	42	9.9 (2.9)	9.7 (5.9–15.3)	↑	0.2–0.7
lactate v (mmol/L)	43	11.6 (2.7)	11.4 (3–16.8)	↑	0.2–0.7
LDH v (U/L)	45	500 (125)	449 (327–862)		225–700

Results of blood gas analysis, electrolytes, and metabolic variables (total protein (Tp), partial pressure of carbon dioxide (pCO_2_), partial pressure of oxygen (pO_2_), bicarbonate (HCO_3_^−^), base excess (BE), packed cell volume (PCV), sodium (Na^+^), potassium (K^+^), calcium (Ca^2+^), chloride (Cl^−^), glucose, lactate, and lactate dehydrogenase (LDH)) of arterial (a) and venous (v) samples taken at 0, 60, 120, 180, and 240 min. Sample size (N), mean, standard derivation (SD), median, range, and reference intervals are presented. A maximum of 45 samples was used where all samples of all specimens were available, otherwise this number is smaller. Reference intervals are shown in the right column [18] and statistically significant (*P* < 0.05) differences are marked with an arrow up if they are higher or down if they are lower than reference intervals

**Fig. 2:**
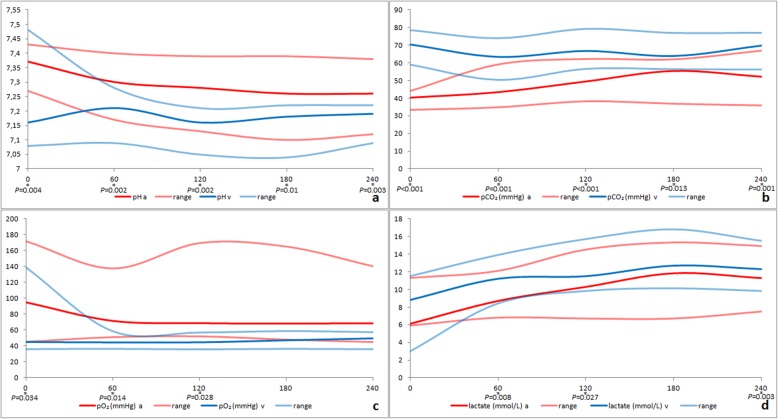
Line chart from median and range of arterial a and venous (v) values of *a* pH *b* partial pressure of carbon dioxide (pCO_2_) *c* partial pressure of oxygen (pO_2_) and *d* lactate at 0, 60, 120, 180, and 240 min perfusion. Significant differences between venous and arterial values are marked with asterisks, and *p*-values are reported

In order to evaluate metabolism of ileum specimens at each time point, parameters of arterial and venous blood samples were compared, and significant differences were noted (Fig. 2, Table 2).

**Table 2:** Median and range of arterial (a) and venous (v) blood parameters (bicarbonate (HCO_3_^−^), base excess (BE), sodium (Na^+^), potassium (K^+^), calcium (Ca^2+^), chloride (Cl^−^), and glucose) at 0, 60, 120, 180, and 240 min perfusion, *p*-values are reported if arterial and venous blood parameters are significantly different

time (min)	0		60		120		180		240	
	median (range)	*P*	median (range)	*P*	median (range)	*P*	median (range)	*P*	median (range)	*P*
HCO3- a (mmol/L)	22.8 (19.3–24.5)	0.003	21.1 (20.3–25)	0.011	22.7 (19.7–25.5)	0.023	22.6 (20.7–25.2)	0.033	22.2 (19.9–27.1)	
HCO3- v (mmol/L)	23.6 (21.4–24.7)		24.2 (21–25.1)		23.5 (21.4–26.6)		22.4 (22.3–26.4)		22.9 (21.4–29.9)	
BE a (mmol/L)	−2.3 (−7.6--0.9)	<0.001	−3.9 (−8.3--2.3)		−3.5 (−9.3--2)		−5 (−6.1--0.8)		−2.7 (−8.2–0.1)	
BE v (mmol/L)	−5.3 (−7.4--4.4)		−3.8 (−8.4--2.9)		−4.4 (−9--1.6)		−4.9 (− 7.5--2.2)		− 5.1 (− 7.3--1.3)	
Na + a (mmol/L)	135.3 (132–138.8)	0.001	138.9 (132.6–140.5)	<0.001	139 (131.6–141.7)	<0.001	140.65 (135.1–152)	0.002	138 (137.2–146)	0.001
Na + v (mmol/L)	140.6 (134.8–144)		143.5 (138.7–144.3)		143.6 (138.7–148.2)		145.8 (137.2–154)		146.5 (140–152)	
K+ a (mmol/L)	5.76 (4.66–6.8)		5.61 (4.91–6.43)		5.73 (4.46–6.19)		5.62 (4.47–6.95)		5.86 (4.71–6.89)	0.049
K+ v (mmol/L)	6.43 (5.05–9.6)		5.49 (4.46–7.09)		5.54 (4.46–6.55)		5.76 (4.66–7.18)		6.6 (4.91–7.6)	
Ca2+ a (mmol/L)	1.48 (1.4–1.62)		1.52 (1.41–1.59)		1.5 (1.41–1.62)	0.03	1.53 (1.43–1.63)	0.017	1.53 (1.44–1.68)	0.012
Ca2+ v (mmol/L)	1.49 (1.42–1.59)		1.51 (1.44–1.61)		1.53 (1.44–1.63)		1.55 (1.47–1.63)		1.56 (1.46–1.68)	
Cl- a (mmol/L)	105.8 (102.2–108.8)	0.001	107.2 (101.7–110.3)	0.001	109.4 (101.6–114)		107.6 (101.3–113)	0.006	107.6 (101.9–111.5)	0.003
Cl- v (mmol/L)	110.4 (106.5–114.2)		109.3 (102.6–112.6)		109.9 (101.8–113.8)		111.1 (102.1–114.1)		110.9 (101.5–113.2)	
glucose a (mmol/L)	85 (76–105)	0.005	116 (93–141)	<0.001	112 (95–187)	0.002	121 (85–190)	0.019	105 (92–176)	0.026
glucose v (mmol/L)	162 (126–339)		136 (117–178)		142 (111–208)		145 (107–219)		121 (100–201)	

**Fig. 3:**
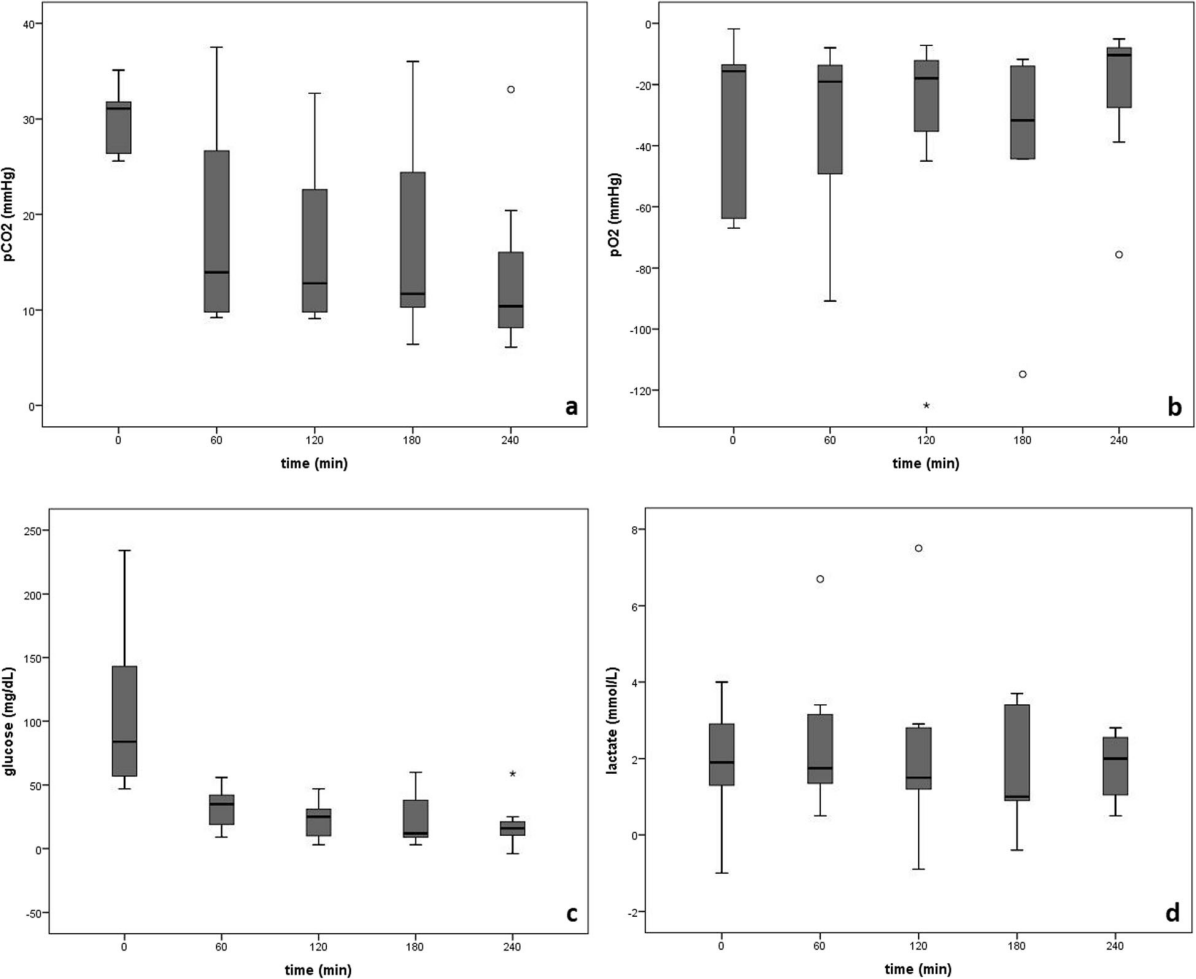
Box-and-whisker plot of the calculated differences between venous and arterial values of *a* partial pressure of carbon dioxide (pCO_2_) *b* partial pressure of oxygen (pO_2_) *c* glucose and *d* lactate at 0, 60, 120, 180, and 240 min perfusion. Boxes represent the quartiles, lines within the boxes the median, whiskers show the minimum and maximum except for outliers, which are plotted as individual points if 1.5× outside the quartile range or asterisks if 3x outside the quartile range. Quartiles of calculated differences between venous and arterial values are positive for pCO_2_, glucose and lactate and negative for pO_2_

In order to document changes in metabolism of ileum specimens over time, blood parameters and the calculated differences between venous and arterial parameters were compared between the start of perfusion to 60 min, 120 min, 180 min, and 240 min perfusion time. There was no significant difference over time compared to baseline values for the calculated difference between venous and arterial pH, pO_2_, HCO_3_^−^, PCV, Na^+^, K^+^, Ca^2+^, lactate, but glucose decreased at 240 min (*P* = 0.047). Calculated differences decreased significantly over time between venous and arterial pCO_2_ (*P* = 0.001) and Cl^−^ (*P* = 0.012; except at 240 min) and BE increased significantly (*P* < 0.001) over time. Arterial pCO_2_ values increased significantly (*P* = 0.001) over time. Calculated differences between venous and arterial pO_2_, pCO_2_, glucose, and lactate are presented in Fig. 3. There was no significant difference for LDH and Tp over time compared to baseline values.

### Pathology

Gross pathology showed a mild to moderate reddening of mucosa with largely intact mucosal lining.

**Fig. 4:**
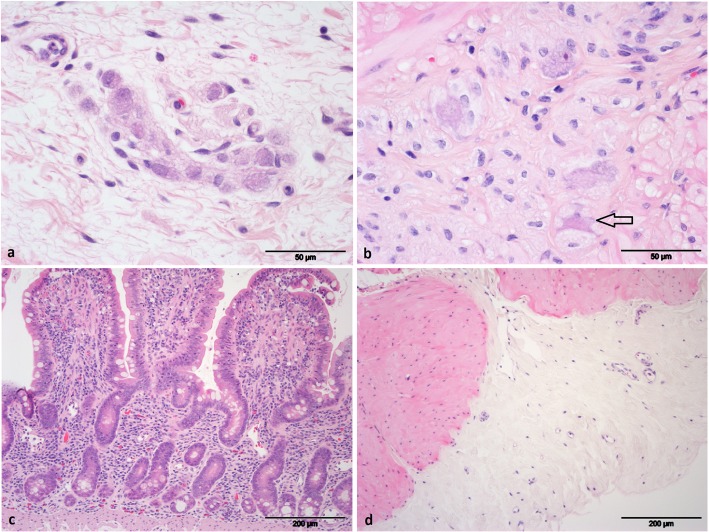
Haematoxylin and eosin stained slides of ileum biopsies after 4 h of perfusion showing *a* neurons of the submucosal plexus (× 400 magnification) - all neurons are normal *b* neurons of the myenteric plexus (× 400 magnification) - 3 normal neurons, 1 poorly preserved (arrow) *c* mucosa (× 100 magnification) *d* serosa (× 100 magnification)

Biopsy sections were overall well preserved with variable loss of surface epithelium at the tips of villi and normal epithelial cells in the body and crypts of villi. Neurons in the submucosal plexus (SMP) were well preserved, with 99% (±1%) showing normal features. Nissl substance, nucleoli and normal cytoplasmic staining was found in the majority of neurons. Subtly increased cytoplasmic vacuolation in a few cases was the only abnormality. Some neurons within the myenteric plexus (MP) were less well preserved with 76.1% (±23.9%) showing normal features. Eosinophilic cytoplasm and shrunken neurons with eccentric nuclei were present in those classified as abnormal. There was no significant difference between the biopsy scoring of the perfusion with the addition of methylprednisolone and without. Detailed results are shown in Table 3 and examples of histological sections are shown in Fig. 4.

**Table 3:** Histological scoring of biopsies

	Perfused samples of submucosal plexus				Control samples of submucosal plexus			
Ileum	normal	degenerate	% normal	% degenerate	normal	degenerate	% normal	% degenerate
1	185	1	99.5	0.5	146	0	100	0
2	75	1	98.7	1.3	36	0	100	0
3	68	0	100	0	47	2	95.9	4.1
4	106	0	100	0	26	1	96.3	3.7
5	27	0	100	0	21	0	100	0
6	83	0	100	0	32	0	100	0
7	63	0	100	0	22	0	100	0
8	59	0	100	0	92	0	100	0
9	25	2	92.6	7.4	31	0	100	0
	Perfused samples of myenteric plexus				Control samples of myenteric plexus			
	normal	degenerate	% normal	% degenerate	normal	degenerate	% normal	% degenerate
1	11	3	78.6	21.4	23	3	88.5	11.5
2	39	16	70.9	29.1	25	7	78.1	21.9
3	19	4	82.6	17.4	7	1	87.5	12.5
4	20	16	55.6	44.4	27	12	69.2	30.8
5	18	0	100	0	26	1	96.3	3.7
6	43	8	84.3	15.7	2	2	50	50
7	5	0	100	0	20	7	74.1	25.9
8	14	2	87.5	12.5	13	5	72.2	27.8
9	3	9	25	75	39	5	88.6	11.4

Neuron counts and percentages of normal and degenerate neurons in the submucosal plexus and myenteric plexus of post perfusion biopsies and control biopsies (taken fresh, before transport and perfusion) per section; each of the 9 ilei is listed. There were no significant differences in percentages of normal and degenerate neurons between perfused samples and control samples

### Controls

Two of the control specimens showed subtle motility after 25 min and 110 min, which stopped 55 min and 50 min later, respectively. The colour of the serosa remained pale in all control specimens for the duration of the experiment. Mass of the control ilei after the experiment was between 88.9 and 185.7% (mean 106.2 ± 30%) of its original mass. The increase in mass was not significantly different to the perfusion ilei. Macroscopically, the mucosa appeared autolytic and was malodorous. Similar to the biopsies taken after perfusion, control biopsies showed 99.1% (±1.7%) normal neurons in the SMP and 78.3 (±14%) in the MP. Detailed results are shown in Table 3.

## Discussion

In the present study, isolated equine ileum perfusion was established as a new model to investigate equine intestinal disease. This ileum perfusion was adapted from the isolated equine limb perfusion, and from this experience, a sample size of 9 horses was chosen and the statistically significant differences, especially of arterial compared to venous blood parameters, show that this sample size was adequate to describe the effects of perfusion on equine ileum [12]. In contrast to the limb, the ileum is a highly ischemia-sensitive organ [19] and the detrimental effects of warm ischemia have been shown in mice after only 35 min [20]. To improve the outcome of the perfusion experiment, methylprednisolone was used to attenuate the effects of reperfusion injury [21, 22]. In our study, no significant differences in histology could be found between specimens with and without this medication, but sample size might be too small to detect subtle differences.

Due to the increase in blood flow during the equilibration period, the blood pressure increased significantly and reached physiological values [23], except for the first two experiments, where technical difficulties led to reduced blood flow during the perfusion; this explains the reported minimum blood pressure values. After that, pressure did not change significantly over time, similar to perfusion of equine limbs [12]. The continuous motility of the ilei and the pink to red colour of the serosa during the experiment suggests good viability of the organ. In 4 experiments the ilei turned cyanotic towards the end, this could be due to suboptimal oxygenation. Studies with a technically more advanced artificial lung may reduce this problem. Mass gain of perfused organs is used as an index for oedema formation and has been reported from around 1% in limbs [12, 24] up to around 30% in porcine free muscle flaps [25]. Perfusion of pig duodenum for 160 min resulted in a mass gain up to 10% [26]. In contrast to other organs, the mucosa of the ileum does produce mucus. This would lead to a higher mass gain, which could not be attributed to oedema formation. To counter this, the content was carefully removed from the lumen after perfusion; this was also done before the start of the perfusion. However, any differences in how successfully the content could be removed may have created some of the changes in mass; this is most likely the reason for the loss of ileal mass but it should also be considered when interpreting the noted mass gain. The high variance in mass gain is also reflected in the results of mass gain of control ilei. Mean values of 5% mass gain reported in the present study may therefore not fully reflect true values, even though they are in a range comparable to other studies [25, 26].

Significant differences between arterial compared to venous blood parameters show the functional metabolism of the perfused organ. The oxygen consumption and carbon dioxide production of the tissues is documented by the significant differences between arterial and venous values. Also, there was very little change in the calculated differences between venous and arterial blood parameters throughout the experiments, which may be a sign of the stability of the method over this time period. Arterial and venous lactate concentrations were significantly increased, partly due to the lack of the lactate-metabolizing-effect of the liver [24], and as a consequence, arterial and venous pH was lower than reference intervals. Lactate concentrations in the initial arterial blood samples were also higher than reference intervals; this leads to the conclusion that besides the partially anaerobic metabolism of the perfused organ, the blood itself also shows anaerobic metabolism; this is in accordance with human blood transfusions, where increasing lactate concentrations depend on the storage time [27]. High venous pCO_2_ and lactate concentrations also led to the decrease of venous pH, BE, and HCO_3_^−^ compared to reference intervals. The mild decrease of the calculated difference between venous and arterial pCO_2_ values over time was mainly due to increased arterial pCO_2_ values with the consequence of an increased calculated difference between venous and arterial BE values [28]. Compared to reference intervals, arterial and venous K^+^ is significantly higher, possibly caused by damage to blood cells. At 240 min venous K^+^ is even significantly higher than arterial K^+^, possibly a sign of commencement of cell damage in the perfused organ at this time point [27]. Increased venous glucose as well as Na^+^, Ca^2+^, and Cl^−^ compared to arterial samples demonstrate the uptake through the serosa of the ileum either via diffusion or active uptake by mesothelial cells out of the peritoneal replacement fluid [29, 30]. In human dialysis patients, glucose uptake from dialysis solution decreases over time, with 50 and 75% of glucose uptake within 180 min and 360 min respectively; this may explain the significant decrease of glucose uptake after 240 min in this study [30]. Perfusion of the organ did not lead to haemolysis as demonstrated by the lack of significant difference between arterial compared to venous values of PCV and their difference over time.

Biopsy histopathology results were largely similar to healthy ileum samples with neuronal damage of 1% in the SMP (compared to 0.9% in control biopsies) and 23.9% in the MP (compared to 21.7% in control biopsies). This difference in neuronal degeneration could be linked to normal aging, since loss of neurofilament positive neurons is reported to be 36.07% of the SMP in contrast to 58.04% of the MP in elderly humans compared to young humans [31]. Biopsy results are clearly distinct from EGS biopsies with reported neuronal damage in acute EGS jejunum of 69% for the SMP and 85.2% for the MP [32]. In another study, numbers of normal and damaged neurons per section are reported for the ileum, but these numbers are not directly comparable to the results reported in the present study. However in that study, it is stated that all types of EGS had severe neuronal damage in the ileum, especially in the SMP [33]; whereas in the present study there were hardly any abnormal neurons in the SMP.

This model is limited by its current time frame of 4 hours, as only toxic changes developing within this time may be detected. This duration of experiments was chosen for the present paper, but an increase in perfusion time to 6–8 h or more, as is achievable in e.g. equine distal limbs [12], will be tested. Furthermore, the ileum specimens might have an impaired microbiome, as during preparation of ileum specimens, ileal contents were removed. Also, it is possible that during that step, air entered the intestinal lumen creating an aerobic environment. Toxins that require co-metabolism of the original intestinal microbiota might therefore not show any effect. Beside these limitations, this model could be used in future studies to test if putative toxins induce changes similar to EGS. Furthermore, we propose that the now established model may be useful to test the capacity of agents (such as allopurinol, superoxide dismutase, catalase, or deferoxamine) to minimize I/R injury in the horse, for this question the time frame of perfusion of the present study may already be adequate [3]. Similarly, the pathogenesis of equine POI can be investigated in this model, as well as pharmaceuticals that potentially enhance intestinal motility. Hormonal studies may be performed without the influence of endocrine glands, this could be of benefit for specific research questions. Furthermore, novel pharmaceuticals could be screened for their enteric toxicity before being used in the life horse. Future studies will show the feasibility of these applications, but a wide range of uses seems possible.

## Conclusions

Perfusion of equine ileum obtained from horses slaughtered for meat production for 4 hours is a cost-efficient method that yields viable tissues and enteric neurons. In future studies, this method may be used to test possible causative toxins in healthy ilei for the induction of changes similar to EGS, to investigate preventive pharmaceuticals against I/R injury, and to explore pathogenesis of POI, amongst others.

## Methods

### Pilot studies

Pilot studies were conducted beforehand to develop a repeatable method. Preparation techniques, the perfusion procedure as such and perfusion time were adapted in these experiments.

### Animals

Ileum specimens used in this study were obtained from horses aged 1 year or older routinely slaughtered for commercial meat production. Horses were briefly assessed prior to slaughter and, at this stage, the exclusion criteria for this study were signs of diarrhoea or a body condition score below 4/5. Horses were stunned using a penetrating captive bolt and then killed by exsanguination after severing the jugular and carotid vessels unilaterally. Following death, the horse underwent standard slaughter procedures. After evisceration the stomach and intestines the ileum was obtained from the personnel.

### Preparation of perfusate and ileum

During exsanguination 8 L to 10 L of blood was openly collected in plastic containers and heparin (Heparin Gilvasan, 5000 U/L)^1^ was added for anticoagulation. The blood was then put on ice for transport to the laboratory.

**Fig. 5:**
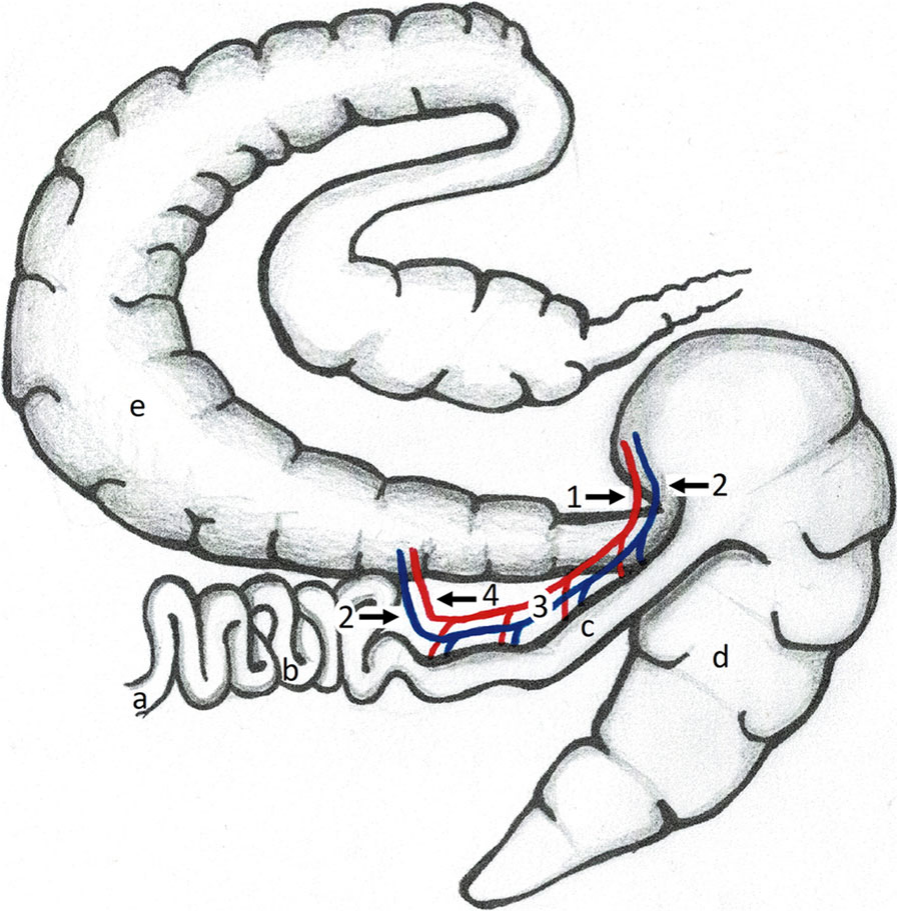
Anatomical illustration of cannulated vessels. *a* duodenum *b* jejunum *c* ileum *d* caecum *e* colon *1* location of arterial cannulation *2* location of venous cannulation *3* ileal artery and vein *4* location of ligation of the ileal artery

The ileum was obtained with the supplying vessels and the adjacent mesentery, and any contents were gently milked out. The aboral artery and vein and the oral vein were cannulated (Fig. 5) with a polyurethane catheter (Extended Use MILACATH®, 12 to 20 Gauge),^2^ using the largest fitting size. For large scale veins, the cut end of a medical-grade polyvinyl chloride tubing (Heidelberger extension, 3 mm internal diameter)^3^ was used. These catheters were secured with a Chinese finger-trap suture. All other visible vessels were ligated. The blood within the ileal vessels was then flushed out with 4 × 50 mL cold isotonic, hyperalbuminaemic, normoglycaemic preservation fluid [12]. After the first 3 perfusions, methylprednisolone (Urbason®, 60 mg/dL)^4^ was added to the final 50 mL preservation fluid [22]. Warm ischemia time was recorded and defined as the time between stunning of the horse and flushing of the ileal vessels.

An endotracheal tube (Rüschelit® Super safety clear, inner diameter 7.5 mm)^5^ was inserted into the aboral part of the ileal lumen and secured with a purse-string suture. The lumen was flushed through this tube with 200 mL of Ringer’s solution (Ringer B. Braun)^3^ and the oral end was then closed with a purse-string suture. The tube was covered by a rubber balloon. The ileum was put into a plastic bag with 1 L peritoneal replacement fluid, containing Na^+^ (146.26 mmol/L), K^+^ (4 mmol/L), Ca^2+^ (2.22 mmol/L), Cl^−^ (154.76 mmol/L), HCO_3_^−^ (35.05 mmol/L), and glucose (154.5 mg/dL) [34] and kept on ice for transport.

### Perfusion

The perfusate was prepared by mixing autologous plasma and blood in a 2:3 ratio [12]. Perfusate that was not immediately used for perfusion was stored at 4 °C and, if not used to replenish the reservoir, discarded after the experiment.

**Fig. 6:**
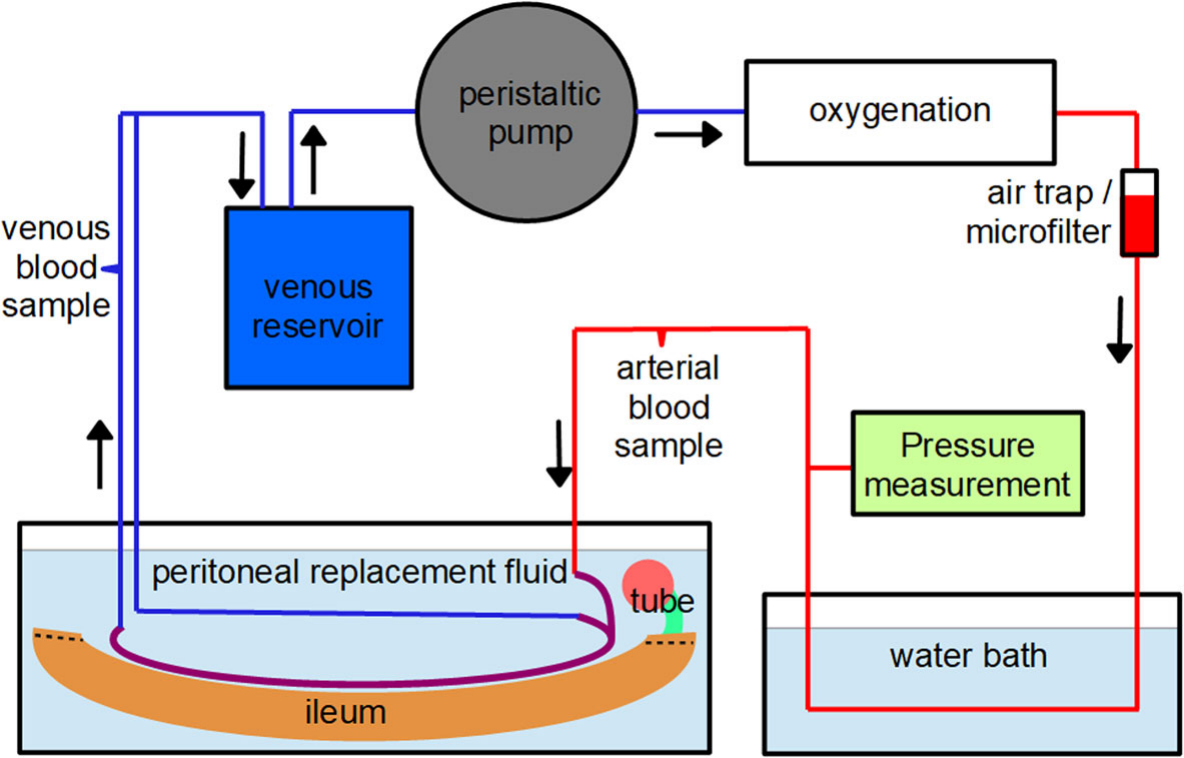
Perfusate is delivered with a peristaltic pump from the venous reservoir through the oxygenation chamber, microfilter and water bath to the ileum specimen and is then collected in the former venous reservoir

Cold ischemia time was recorded and defined as the time period from the flushing of the ileal vessels to the start of perfusion. For the extracorporeal perfusion of the ileum, a recirculating perfusion system was used (Fig. 6). The venous reservoir consisted of 500 mL perfusate on a magnetic stirrer (Variomag® mono)^6^ and was replenished as needed. A peristaltic pump (Masterflex® L/S)^7^ was used to pump the perfusate through medical-grade polyvinyl chloride tubing. Oxygenation was achieved by thin-walled, gas permeable silicone tubing (2 mm internal diameter and 0.25 mm wall thickness)^8^ coiled inside an oxygenation chamber filled with a mixture of oxygen and room air [35]. Microaggregates and air were removed by a microfilter (Transfusion set, pore size 200 μm).^9^ Tubing was then directed through a water bath set at 37.0–37.5 °C [36]. Arterial pressure was measured continuously with a digital pressure measurement instrument (Manometer GDH 200–13)^10^ and recorded every 10 min. The ileum was connected to the circuit using the described arterial and venous catheters and placed in a bath filled with warm peritoneal replacement fluid (37.0–37.5 °C) [36].

Total perfusion time was 4 h, including a 30 min equilibration period. During this equilibration period, the blood flow was started with 3.5–6 mL/min depending on the mass of the ileum and increased every 5 min by 0.5–10 mL/min depending on the blood pressure at that time in order to avoid oedema formation as a result of high resistance in the vessels of the cold tissue [12]. To achieve physiological blood flow of 21.4 ± 0.8 mL/min/kg [37], blood flow was set to a minimum of 20 mL/min/kg and further increased to achieve a physiological blood pressures of about 80 mmHg [23]. Motility of the ileum and colour of the serosa was recorded every 10 min. After perfusion, the mucus produced by the ileum was again gently milked out using manual compression. To calculate changes in tissue mass, the ileum was weighed before and after perfusion [12].

### Control ilei

At the abattoir, the oral third of the ileum was separated and used as a control. The control part underwent the same temperature changes during transport and perfusion and was also kept in peritoneal replacement fluid, but it did not undergo preparation, flushing or perfusion. Before and after the experiment, the control part was weighed. Gross pathology was performed after the experiment similar to the perfused ileum.

### Perfusate sampling

Arterial and venous samples of the perfusate were taken before and after flowing through the ileum respectively at the beginning and then at 1-h intervals through two 3-way valves (Discofix® 3)^3^. Samples were analysed immediately by a blood gas analyser (Cobas® b123)^11^ and glucose, lactate, pCO_2_, pO_2_, pH, HCO_3_^−^, BE, PCV, Na^+^, K^+^, Ca^2+^, and Cl^−^ were documented.

For the evaluation of LDH and Tp, an additional venous blood sample was taken every hour and centrifuged (Hettich® EBA 20)^12^ at 3000 rpm for 5 min. The plasma was immediately separated, Tp measured by an optical refractometer (RHC-200 ATC)^13^ and the remaining plasma stored at 4 °C for 12–24 h before being analysed for LDH (Cobas® c501)^11^ at a clinical laboratory certified by the International Organization for Standardization 9001:2008.

### Pathology

Full thickness biopsies sized approximately 1 × 1 cm were taken from the ileum before preparation (as fresh controls) and after perfusion. After perfusion, the ileum vessels were flushed with warm preservation solution and gross pathology was performed by opening the ileum longitudinally to examine the mucosa with final biopsies taken from the middle of the ileum.

Biopsies were stored in 4% buffered formalin for fixation. Specimens were embedded in paraffin, stained with haematoxylin and eosin and evaluated using light microscopy (EM). The sections were assessed for general preservation and preservation of neurons in particular. Based on their morphology, healthy and degenerate neurons in the SMP and the MP were counted. Degeneration was defined as eosinophilic cytoplasm, loss of Nissl substance, pyknotic nucleus or severe shrinkage.

### Statistical analysis

Statistical analysis was performed with a commercial software program (IBM SPSS Statistics 24).^14^ Normal distribution was tested with a Kolmogorov-Smirnov test. Blood samples taken at 0 min were set as a baseline to compare calculated differences between venous and arterial values over time. Arterial pCO_2_ values were compared over time in the same way to evaluate the performance of the artificial lung. Mixed model analysis was performed to compare results of blood samples followed by pairwise comparisons using Sidak’s alpha correction procedure for significant differences between arterial compared to venous blood samples and of calculated differences between venous and arterial values over time compared to start of perfusion. Arterial and venous blood parameters were compared to laboratory reference intervals by calculating a confidence interval of 95%; if the lower limit of the confidence interval was outside upper reference intervals, the parameter was counted as significantly higher and if the upper limit of the confidence interval was outside lower reference intervals, the parameter was counted as significantly lower than reference intervals. Additionally baseline lactate concentrations (taken at 0 min) were compared to reference intervals in the same way to distinguish between lactate produced by the ileum and lactate already contained in the perfusate. To compare results of ileum perfusion with and without methylprednisolone, a Mann-Whitney U-test was used. Mass increase between perfusion ilei and control ilei as well as percentage of normal and degenerate neurons in perfused and control biopsies were compared with a Wilcoxon signed rank test. For all statistical analyses, a *p*-value below 5% (*P* < 0.05) was regarded as significant.

## Data Availability

The datasets used and/or analysed during the current study are available from the corresponding author on reasonable request.

## Abbreviations

BE: base excess
Ca^2+^: calcium
Cl^−^: chloride
EGS: Equine Grass Sickness
HCO_3_^−^: bicarbonate
I/R: ischemia/reperfusion
K^+^: potassium
LDH: lactate dehydrogenase
MP: myenteric plexus
Na^+^: sodium
pCO_2_: partial pressure of carbon dioxide
PCV: packed cell volume
pO_2_: partial pressure of oxygen
POI: post-operative ileus
SMP: submucosal plexus
Tp: total protein

## Footnotes

1 Gilvasan Pharma GmbH, Vienna, Austria.

2 MILA International, Inc., Florence, USA.

3 B. Braun, Melsungen, Germany.

4 Sanofi-Aventis, Frankfurt, Germany.

5 Rüsch Austria GmbH, Vienna, Austria.

6 Thermo Fisher Scientific Inc., Waltham, USA.

7 Cole-Parmer Instruments Co Ltd., London, UK.

8 J. Lindemann GmbH, Helmstedt, Germany.

9 Codan Medizinische Geräte GmbH & Co KG, Lensahn, Germany.

10 Greisinger Electronic GmbH, Regenstauf, Germany.

11 Roche Diagnostics GmbH, Vienna, Austria.

12 Andreas Hettich GmbH & Co.KG, Tuttlingen, Germany.

13 Uzman-Versand UG & Co. KG, Bocholt, Germany.

14 IBM Deutschland GmbH, Ehningen, Germany.

## References

[1] Freeman DE (2018). Fifty years of colic surgery. Equine Vet J.

[2] McGorum BC, Pirie RS (2018). Equine Dysautonomia. Vet Clin North Am Equine Pract.

[3] McMichael M, Moore RM (2004). Ischemia-reperfusion injury pathophysiology, part I. J Vet Emerg Crit Care.

[4] Lisowski ZM, Pirie RS, Blikslager AT, Lefebvre D, Hume DA, Hudson NPH (2018). An update on equine post-operative ileus: definitions, pathophysiology and management. Equine Vet J.

[5] Daniel CR, Labens R, Argyle D, Licka TF (2018). Extracorporeal perfusion of isolated organs of large animals - Bridging the gap between in vitro and in vivo studies. ALTEX.

[6] Hansen L, Hartmann B, Mineo H, Holst JJ (2004). Glucagon-like peptide-1 secretion is influenced by perfusate glucose concentration and by a feedback mechanism involving somatostatin in isolated perfused porcine ileum. Regul Pept.

[7] Hansen L, Hartmann B, Bisgaard T, Mineo H, Jørgensen PN, Holst JJ (2000). Somatostatin restrains the secretion of glucagon-like peptide-1 and -2 from isolated perfused porcine ileum. Am J Physiol Endocrinol Metab.

[8] Rehfeld JF, Holst JJ, Lindkær JS (1982). The molecular nature of vascularly released cholecystokinin from the isolated perfused porcine duodenum. Regul Pept.

[9] Messell T, Harling H, Seier Poulsen S, Bersani M, Holst JJ (1992). Extrinsic control of the release of galanin and VIP from intrinsic nerves of isolated, perfused, porcine ileum. Regul Pept.

[10] Vatistas NJ, Nieto JE, van Hoogmoed L, Gardner I, Snyder JR (2003). Use of an isolated intestinal circuit to evaluate the effect of ischemia and reperfusion on mucosal permeability of the equine jejunum. Vet Surg.

[11] Polyak MMR, Morton AJ, Grosche A, Matyjaszek S, Freeman DE (2008). Effect of a novel solution for organ preservation on equine large colon in an isolated pulsatile perfusion system. Equine Vet J.

[12] Patan B, Budras K-D, Licka TF (2009). Effects of long-term extracorporeal blood perfusion of the distal portion of isolated equine forelimbs on metabolic variables and morphology of laminar tissue. Am J Vet Res.

[13] Patan-Zugaj B, Gauff FC, Plendl J, Licka TF (2014). Effect of endotoxin on leukocyte activation and migration into laminar tissue of isolated perfused equine limbs. Am J Vet Res.

[14] Gauff FC, Patan-Zugaj B, Licka TF (2014). Effect of short-term hyperinsulinemia on the localization and expression of endothelin receptors a and b in lamellar tissue of the forelimbs of horses. Am J Vet Res.

[15] Gauff FC, Patan-Zugaj B, Licka TF (2013). Hyperinsulinaemia increases vascular resistance and endothelin-1 expression in the equine digit. Equine Vet J.

[16] Patan-Zugaj B, Gauff FC, Licka TF (2012). Effects of the addition of endotoxin during perfusion of isolated forelimbs of equine cadavers. Am J Vet Res.

[17] Unterköfler MS, McGorum BC, Milne EM, Licka TF. Establishment of a model for equine grass sickness: Extracorporeal blood perfusion of equine ileum. In: Aulock S von, editor. 18th Annual Congress of EUSAAT; September 23–26, 2018; Linz. Kuesnacht: Society ALTEX Edition; 2018. p. 260.

[18] Corley K (2008). The equine hospital manual.

[19] Muñoz-Abraham AS, Patrón-Lozano R, Narayan RR, Judeeba SS, Alkukhun A, Alfadda TI (2016). Extracorporeal hypothermic perfusion device for intestinal graft preservation to decrease ischemic injury during transportation. J Gastrointest Surg.

[20] Stringa P, Lausada N, Romanin D, Machuca M, Cabanne A, Rumbo M, Gondolesi G (2012). Defining the nonreturn time for intestinal ischemia reperfusion injury in mice. Transplant Proc.

[21] Martens A, Boada M, Vanaudenaerde BM, Verleden SE, Vos R, Verleden GM (2016). Steroids can reduce warm ischemic reperfusion injury in a porcine donation after circulatory death model with ex vivo lung perfusion evaluation. Transpl Int.

[22] Sack F-U, Reidenbach B, Dollner R, Schledt A, Gebhard MM, Hagl S (2001). Influence of steroids on microvascular perfusion injury of the bowel induced by extracorporeal circulation. Ann Thorac Surg.

[23] Hopster K, Hopster-Iversen C, Geburek F, Rohn K, Kästner SBR (2015). Temporal and concentration effects of isoflurane anaesthesia on intestinal tissue oxygenation and perfusion in horses. Vet J.

[24] Constantinescu MA, Knall E, Xu X, Kiermeir DM, Jenni H, Gygax E (2011). Preservation of amputated extremities by extracorporeal blood perfusion; a feasibility study in a porcine model. J Surg Res.

[25] Dragu A, Birkholz T, Kleinmann JA, Schnürer S, Münch F, Cesnjevar R (2011). Extracorporeal perfusion of free muscle flaps in a porcine model using a miniaturized perfusion system. Arch Orthop Trauma Surg.

[26] Holst JJ, Lauritsen K, Jensen SL, Nielsen OV, Schaffalitzky de Muckadell OB (1981). Secretin release from the isolated, vascularly perfused pig duodenum. J Physiol.

[27] Oyet C, Okongo B, Onyuthi RA, Muwanguzi E (2018). Biochemical changes in stored donor units: implications on the efficacy of blood transfusion. J Blood Med.

[28] Muir W, Hubbell JAE (2009). Equine anesthesia: Monitoring and emergency therapy. 2nd ed.

[29] Schröppel B, Fischereder M, Wiese P, Segerer S, Huber S, Kretzler M (1998). Expression of glucose transporters in human peritoneal mesothelial cells. Kidney Int.

[30] Heimbürger O, Waniewski J, Werynski A, Lindholm B (1992). A quantitative description of solute and fluid transport during peritoneal dialysis. Kidney Int.

[31] Xuan Q, Zhang Y-X, Liu D-G, Chan P, Xu S-L, Cui Y-Q (2016). Post-translational modifications of α-synuclein contribute to neurodegeneration in the colon of elderly individuals. Mol Med Rep.

[32] Pogson DM, Doxey DL, Gilmour JS, Milne EM, Chisholm HK (1992). Autonomic neurone degeneration in equine dysautonomia (grass sickness). J Comp Pathol.

[33] Doxey DL, Milne EM, Woodman MP, Gilmour JS, Chisholm HK (1995). Small intestine and small colon neuropathy in equine dysautonomia (grass sickness). Vet Res Commun.

[34] Ross LA, Labato MA (2013). Current techniques in peritoneal dialysis. J Vet Emerg Crit Care.

[35] Hamilton RL, Berry MN, Williams MC, Severinghaus EM (1974). A simple and inexpensive membrane 'lung' for small organ perfusion. J Lipid Res.

[36] Fintl C, Hudson NPH, Handel I, Pearson GT (2016). The effect of temperature changes on in vitro slow wave activity in the equine ileum. Equine Vet J.

[37] Dabareiner RM, White NA, Donaldson LL (2001). Effects of intraluminal distention and decompression on microvascular permeability and hemodynamics of the equine jejunum. Am J Vet Res.

